# Oculomotor deficits in Parkinson's disease: Increasing sensitivity using multivariate approaches

**DOI:** 10.3389/fdgth.2022.939677

**Published:** 2022-08-04

**Authors:** Oliver Bredemeyer, Salil Patel, James J. FitzGerald, Chrystalina A. Antoniades

**Affiliations:** ^1^NeuroMetrology Group, Nuffield Department of Clinical Neurosciences, Medical Sciences Division, University of Oxford, Oxford, United Kingdom; ^2^Nuffield Department of Surgical Sciences, University of Oxford, Oxford, United Kingdom; ^3^Oxford Functional Neurosurgery, John Radcliffe Hospital, Oxford, United Kingdom

**Keywords:** saccades, oculometry, multivariate approach, damping, Parkinson's disease

## Abstract

Parkinson's disease (PD) affects several domains of neurological function, from lower-level motor programs to higher cognitive processing. As certain types of eye movements (saccades) are fast, non-fatiguing, and can be measured objectively and non-invasively, they are a promising candidate for quantifying motor and cognitive dysfunction in PD, as well as other movement disorders. In this pilot study, we evaluate the latency (reaction time), damping (resistance to oscillation), and amplitude of saccadic movements in two tasks performed by 25 PD patients with mild to moderate disease and 26 age-matched healthy controls. As well as general increases in reaction time caused by PD, the damping of saccadic eye movements was found to be task-dependent and affected by disease. Finally, we introduce a proof-of-concept multivariate model to demonstrate how information from saccadometry can be combined to infer disease status.

## Introduction

**Figure 1:**
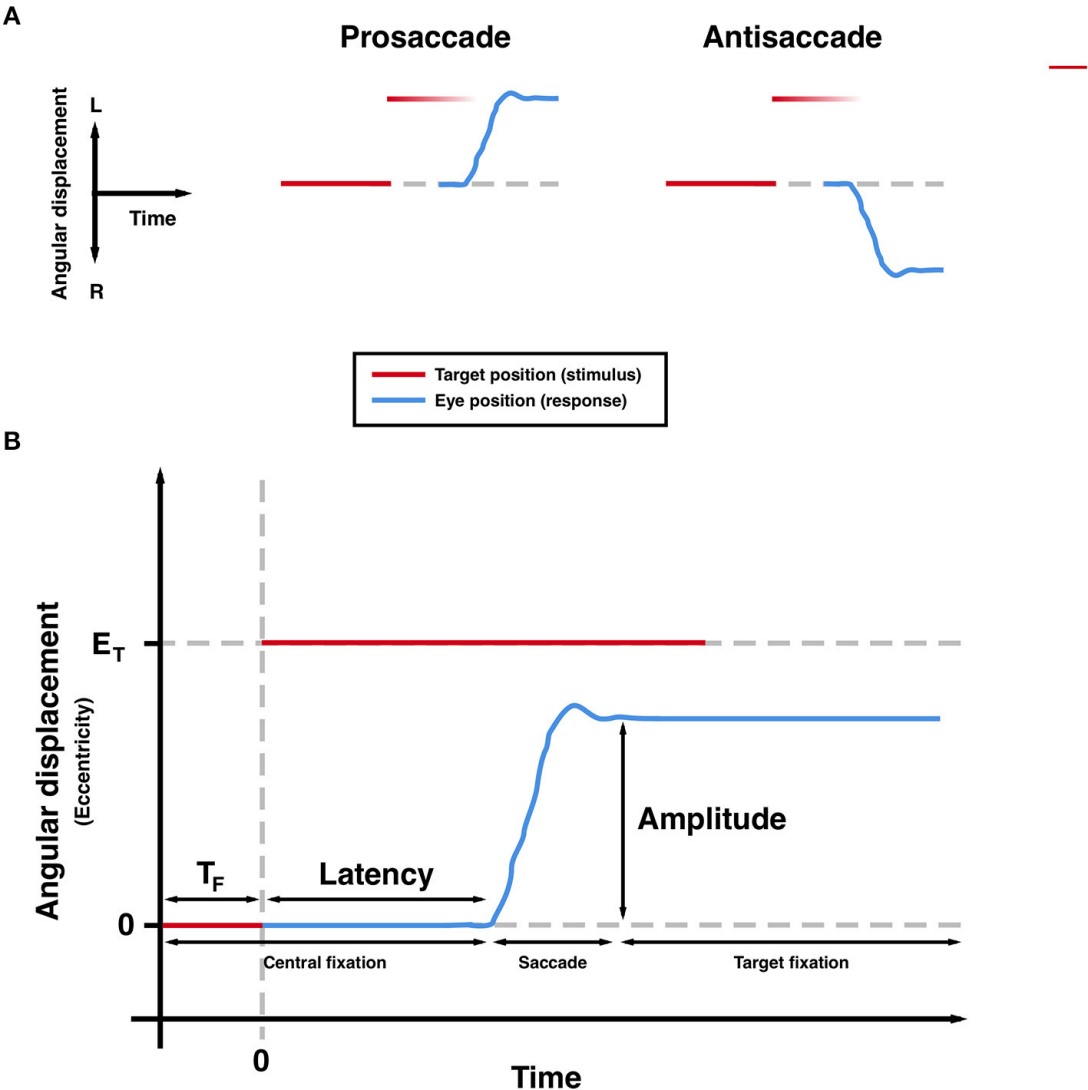
Saccadic paradigms **(A)** and commonly measured parameters **(B)**. *T*_*F*_*, foreperiod; E*_*T*_*, target eccentricity*. Hypometria is observed in the trajectory **(B)**.

The measurement of saccades, the rapid and ballistic movements made by both eyes from one fixation point to another, can provide a highly precise and non-invasive means of quantifying neurological function. A number of brain regions and interconnecting pathways are required to generate appropriate saccades, meaning that tasks can be designed to isolate and probe these structures (1–4). Previous studies have shown characteristic patterns of deficits in these saccadic tasks in patients with movement disorders such as Parkinson's disease (PD), reflecting underlying dysfunction in the neural networks required to generate appropriate saccadic responses. Large meta-analyses have confirmed increased latencies to response in both the prosaccade (PS) and anti-saccade (AS) tasks (see Figure 1) in patients with PD (5, 6). Additionally, more recent findings suggest that latency and other metrics are sensitive to treatment (7–9) and longitudinal change (10).

This body of research sets the stage for the development of robust and clinically useful quantitative measures of motor and cognitive function in movement disorders such as PD. Successful implementations could supplement existing clinical rating scales such as the Movement Disorder Society- Unified Parkinson's Disease Rating Scale (MDS-UPDRS), identify subgroups among existing clinical phenotypes for better stratification of participants in clinical trials, and potentially act as screening tools if suitably specific for disease.

However, signals in saccade metrics can be weak and sensitive to experimental conditions, leading to variability between studies where protocols are insufficiently standardized (5) and presenting a significant barrier to their implementation in clinical practice. This can be addressed by three approaches: standardization of protocols, the derivation of new metrics which are more sensitive to the presence and progression of disease, and multivariate approaches to analysis which can identify subtle patterns of deviation across many parameters.

To that end, standardized protocols have been developed for delivering batteries of tasks under consistent experimental conditions and measuring elicited saccades in high resolution (11, 12). Additionally, with sufficiently high-resolution measurement of saccadic trajectories, a number of new metrics could be derived directly from existing datasets. For example, although rarely used, a previous small cross-sectional study identified saccadic damping as a potential sensitive indicator of PD which may change with progression (13). Damping quantifies the resistance of a system to oscillations, and in the saccade generating system can thus regulate the balance between spatial resolution in the terminal phase of the saccade and temporal resolution. With lower damping, the trajectory of the saccade crosses the target location earlier, while an increase in damping minimizes retinal slip after the target is reached.

In this study we attempt to address each of the three approaches described, using data from a large-scale prospective cohort study. In order to evaluate the task- and disease-dependence of saccadic damping, we calculated this directly from raw data gathered in a high resolution during execution of a standardized protocol. Additionally, we outline a multivariate approach to analysis which aims to identify patterns of deviation amongst our patient groups.

## Materials and methods

### Participants

This work was conducted as part of the ongoing Oxford Quantification in Parkinsonism (OxQUIP) study, a large-scale prospective cohort study based at the John Radcliffe Hospital (Oxford, UK). The study has been approved by the local ethics committee (Research Ethics Committee reference 16/SW/0262), and experiments were conducted in accordance with ICH Good Clinical Practice guidelines and the Declaration of Helsinki.

We recruited 25 patients with Parkinson's disease (13 male, 12 female), and 26 sex-matched healthy controls (13 male, 13 female). The groups were also age-matched (mean age of groups 66 and 69 years respectively, two tailed *t-test p* = 0.14). Patients in the PD group had clinically probable idiopathic PD as determined by the UK Parkinson's Disease Society Brain Bank criteria (14), and at the time of testing, patients had a disease duration of no more than 8 years since diagnosis. Patients receiving deep brain stimulation were excluded from this study. All testing of PD patients was carried out in a well-defined ‘on' state, 60 min after their usual dose of levodopa and other antiparkinsonian medications.

### Saccadometry

Saccadometry was conducted as per a previously published internationally standardized protocol (11), implemented using a head-mounted device (Saccadometer Advanced; Ober Consulting, Poznan, Poland). Targets were projected by integrated lasers onto a plain, matte surface in a horizontal line at 0° (fixation target) and ±10° (peripheral targets). During each trial, the fixation target was illuminated for a random foreperiod of 1.0–2.0 s drawn from a truncated exponential distribution, followed by illumination of either the left or right peripheral target (selected randomly with equal probability). Onset of the peripheral target was synchronous with offset of the fixation target (step paradigm; see Figure 1). Saccades were detected online by the Saccadometer Advanced and the lateral target was extinguished 200 ms after the end of a saccadic response to a trial. For prosaccade blocks, participants were instructed to look at the new target position; for antisaccade blocks, participants were instructed to look in the opposite direction.

Saccades contaminated by eye blinks and head movements were detected online and removed from analysis by the Saccadometer Advanced. For all other saccades, calibrated samples of eye position were recorded every 5 ms, from 25 ms before initiation to 20 ms after termination of the saccade, with the saccade defined as the period of time for which velocity exceeded 5°/ s. Additionally, the direction, latency from target presentation to initiation, amplitude, and peak velocity were reported.

### Pre-processing and sanitization

The data were processed using Python (version 3.9) with the Pandas, NumPy, and SciPy packages. In addition to those removed online during recording, all saccades meeting any of the following criteria were excluded from analysis due to likely recording error: latency <100 ms or >1,000 ms, amplitude >40°, peak velocity > 1,000 deg/s.

### Damping ratio estimation

Following the method of Chen et al. (13), saccade trajectories were fitted directly to the position profile of the step response of a second-order system. Each saccade trajectory from time of initiation onwards was fitted independently to the analytic form of the position profile (Equation 1) by sum-of-squares minimization:


(1) $$\hat{\phi }(t+\delta t)=\{\begin{array}{ll} \text{H}(t)A\left(1-\frac{1}{\sqrt{1-\sigma^{2}}}e^{-\sigma \omega_{0}t}\text{Sin}\left(\omega_{0}\sqrt{1-\sigma^{2}t}+\text{acos}\sigma \right)\right), & \sigma <1\\ \text{H}(t)A\left(1-e^{-\omega_{0}t}-\omega_{0}te^{-\omega_{0}t}\right), & \sigma =1\\ \text{H}(t)A\left(1+\frac{\left(\sigma -\sqrt{\sigma^{2}-}1\right)\exp\left(-\omega_{0}t\left(\sigma +\sqrt{\sigma^{2}+1}\right)\right)-\left(\sigma +\sqrt{\sigma^{2}+1}\right)\exp\left(-\omega_{0}t\left(\sigma -\sqrt{\sigma^{2}-1}\right)\right)}{2\sqrt{\sigma^{2}-1}}\right), & \sigma >1 \end{array}$$

where $\hat{\phi }$(*t*) is the estimated angular displacement at time *t*, H is the Heaviside step function, *A* is the gain, σ is the damping ratio, and ω_0_, natural frequency. The system is underdamped when σ <1, critically damped when σ = 1, and overdamped when σ > 1. Observed trajectories were normalized to amplitude 1 before fitting, and to compensate for the limited resolution of the measuring instrument, time shifts δ*t* of up to ±5 ms were permitted for accurate estimation of the saccade initiation time.

### Single-parameter analyses

Statistical analysis was performed using R [(15), version 4.1]. As participants produced saccades with varying rejection and directional error rates, and distributions of resulting parameters can be highly nonnormal, a generalized linear mixed model (GLMM) was deemed to be most appropriate to deal with these unbalanced data, with model parameters informed by our data and the literature. All models included fixed intercept effects of group (between subjects) and task (prosaccade vs. antisaccade with correct response vs. antisaccade with prosaccadic response; within subjects), a fixed group-by-task interaction effect, and random intercept effects of participant identity, with no specific structure imposed on the covariance matrix.

For latency distributions, which are characteristically highly skewed, a gamma GLMM with identity link function was used in line with previous analyses of reaction time data (16).

In the special case of the identity link function with a Gaussian marginal distribution, the GLMM reduces to a linear mixed model (LMM). These have been shown to be robust to low-level violations of distributional assumptions, including skewness, heteroskedasticity, and high degrees of kurtosis with sufficient sample size (17, 18). As the distribution of estimated damping ratios and amplitudes has not been studied as extensively in the literature, this model family was selected to analyze these variables, with standard transformations applied where necessary. Saccades where the damping ratio could not be estimated with suitable precision (estimated standard error >0.5) were excluded from analysis.

GLMMs were fitted to the data using the R package *lme4* (19), using *all_fit* to generate the best model fit using a variety of optimizers. Model fits were evaluated by visual inspection of heteroskedasticity, influence of observations, collinearity of predictors and for normality of random effects using the *performance* R package (20). For LMMs, normality of Pearson residuals were additionally checked, and skewness and kurtosis of Pearson residual distributions are reported.

The significance of fixed effects was evaluated using *afex* (21). For LMMs, the Kenward-Roger procedure was used as it is robust at our sample sizes (18). For other GLMMs, the likelihood ratio test was used to compare the full model to models with dropped factors; χ^2^ test statistics, *p*-values, and the difference between the Akaike information criteria (AICs) of the two models are reported. Where effects were significant (*p* < 0.05), *emmeans* (22) was used to make *post hoc* pairwise between-group and between-task comparisons, with a Bonferroni correction to false discovery rate 0.05.

The directional error rate in the AS task was compared between groups using a Mann–Whitney *U-*test.

### Classifiers

In order to assess the utility of different metrics and provide proof of concept for a multivariate predictive model, we evaluated the performance of two machine learning models on these data.

For each participant, the median, lower quartile, upper quartile, interquartile range, skewness, and kurtosis of the distributions of PS and AS second-order system fit mean squared error, damping ratio, latency, amplitude, and peak velocity were calculated. Additionally, the directional error rate in the AS task was included. Erroneous prosaccades made during execution of the AS task were excluded during calculation of all features except the AS error rate.

One model was a logistic regression model, with a *L*_2_ regularization term to prevent overfitting. Inputs to the model were transformed using a Yeo-Johnson transformation (23) and scaled to mean 0 and variance 1. Logistic regression models are simple and readily interpretable and perform well when classes are linearly separable. However, they do not perform intrinsic feature selection, so we implemented forward feature selection by residual mutual information, an algorithm described fully by Schaffernicht et al. (24). Feature selection was terminated when the cross-validation area under the receiver operating characteristic curve (ROC-AUC) on the training data peaked.

This was compared with a random forest model, with 400 estimators, which are known to achieve good performance while being robust to noise and avoiding overfitting. Additionally, the random forest model can learn nonlinear decision boundaries and performs feature selection, facilitating comparison between the two models in terms of feature importance.

A large discrepancy between the performance of the two models in favor of the random forest would suggest that the decision boundary is nonlinear due to the existence of different phenotypes within the groups, and that future analyses should avoid using linear models unless these phenotypes are identified.

### Classifier evaluation

Both classifiers were implemented in Python (version 3.9) using Scikit-learn [(25), version 1.0].

A receiver operating characteristic (ROC) curve for each classifier was produced using rankings generated by tournament leave-pair-out cross-validation (TLPO-CV) (26), and the area under the curve was evaluated.

TLPO-CV produces a near-unbiased estimate of the true ROC-AUC of the classifier by comparing the ordering of probability estimates between every possible combination of 2 data points when a model is fitted to all remaining data points. For our data, this procedure involved a total of 1,275 model fits, each using 49 trials (participants), for each classifier tested. However, a method for generating accurate confidence intervals for this form of cross-validation remains to be developed (26). Thus, head-to-head tests for differences in ROC-AUC between the two classifiers were conducted using the 5 × 2 cross-validation combined *F* test first described by Alpaydm (27). This test evaluates differences in performance when 50% of the data are randomly allocated to training and test sets respectively, for 5 different allocations. Thus, the two classifiers were tested for a difference in ROC-AUC using the 5 × 2 cross-validation combined *F-*test (27).

When training logistic regression models, transformation, scaling, and the 5-fold cross-validation used in feature selection was nested within the TLPO cross-validation such that the algorithm was completely naïve to the testing data.

Finally, the 10 most important features selected by each model are reported. Seventy-five overlapping subsets of 2/3 of participants were generated randomly, and both models were trained on each subset. For logistic regression, features were ranked by the proportion of fitted models in which the feature was selected. For random forest models, features were ranked by mean decrease in impurity.

## Results

### Parkinson's disease increases response latency

In total, 9,729 saccades were analyzed, with the PD group contributing 4,509 (46%).

An adequate fit was obtained in the gamma GLMM for latency. Likelihood ratio tests using restricted models revealed significant fixed effects of group (ΔAIC = 2.1, $\chi_{1}^{2}=4.08$, *p* = 0.043), task (ΔAIC = 2,856.6, $\chi_{2}^{2}$ = 2,861, *p* < 0.0001), and group-by-task interaction (ΔAIC = 20.4, $\chi_{2}^{2}=24.4$, *p* < 0.0001).

**Figure 2:**
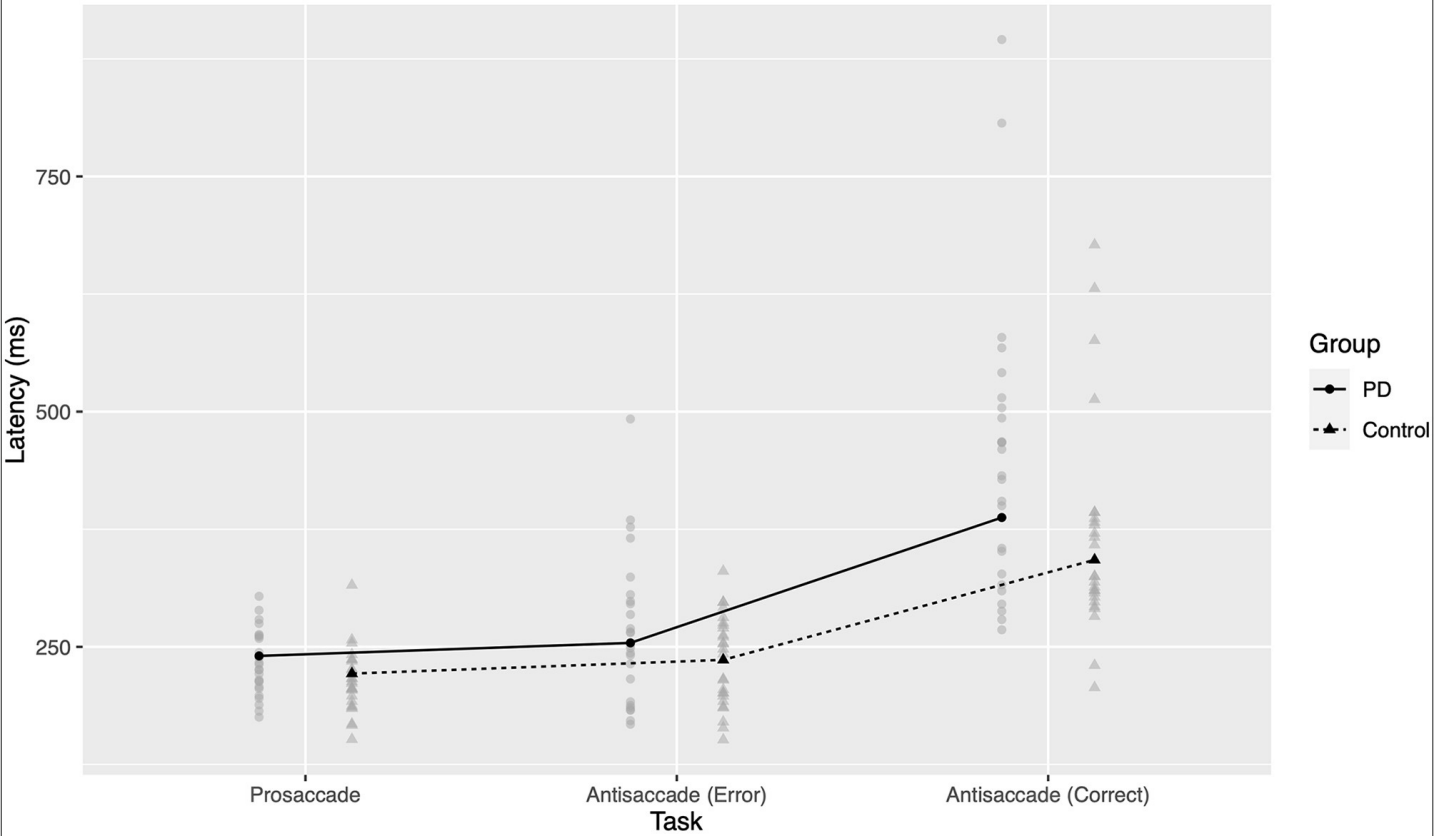
Latency to response across groups and tasks. Gray points represent mean latency for one participant in a given task. Black points and lines represent estimated marginal group means.

*Post-hoc* comparisons were conducted for pairwise within- and between-group differences. Both groups had significant increases in latency from prosaccades, to erroneous prosaccades during the antisaccadic task, to correctly executed antisaccades (all *p* < 0.0001 two-tailed after Bonferroni correction). As seen in Figure 2, the differences in latency between AS errors and correct prosaccades were small (PD 13.9 ms, control 14.6 ms) compared to the differences between correct AS and correct PS (PD 147.1 ms, control 120.9 ms), supporting previous suggestions that these errors arise from a failure of inhibition of a reflexive prosaccade. After Bonferroni correction, there were significant differences in latency between groups in correctly executed antisaccades (PD *c*. 45 ms slower, *p* < 0.0001 two-tailed) and correctly executed prosaccades (PD *c*. 19 ms slower, *p* = 0.046 two-tailed). The effect size for AS errors was similar to that for correct PS but was not significant at the 0.05 level after correction (*p* = 0.069 two-tailed); it is likely that an underlying effect was present but this study did not have sufficient power in this case.

### Variations in damping between saccadic tasks

The residual distribution was somewhat positively skewed (skewness 1.09) and leptokurtic (excess kurtosis 7.8). The LMM was chosen for its robustness to non-normal distributions at sufficient sample sizes (17, 18), but under-dispersion of residuals indicates the analysis may have lost some power.

**Figure 3:**
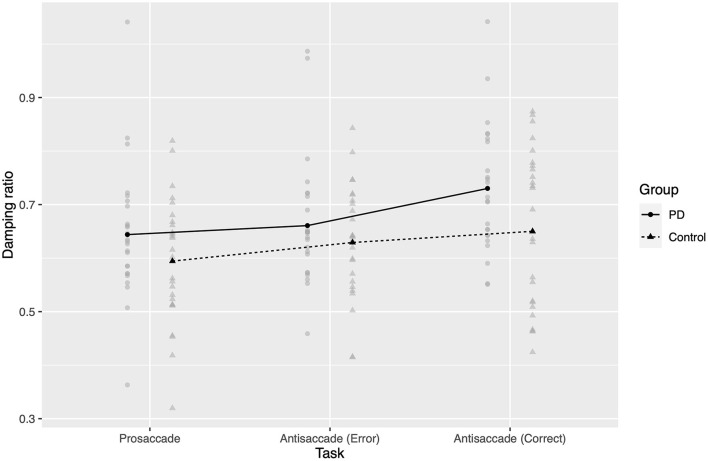
Estimated damping ratios. Gray points represent mean estimated damping ratio for one participant in a given task. Black points and lines represent estimated marginal group means.

Using the Kenward–Roger procedure, damping ratio (Figure 3) was significantly affected by task (*F*_2,9401_ = 98.5, *p* < 0.0001), and there was a significant group-by-task interaction (*F*_2,9401_ = 7.85, *p* = 0.0004). The fixed effect of group was not significant (*F*_1, 49.3_ = 3.43, *p* = 0.07), but was in the same direction as the effect reported by (13).

In *post hoc* comparisons, damping ratio again increased from prosaccades to antisaccades (PD 0.09 greater; control 0.06 greater; both groups *p* < 0.0001 two-tailed after Bonferroni correction). Interestingly, in PD patients the AS errors were more similarly damped to prosaccades (correct vs. error AS 0.07 greater, *p* < 0.0001; error AS vs. correct PS 0.02 greater, *p* = 0.25), while they were more similar to antisaccades in controls (correct vs. error AS 0.02 greater, *p* = 0.13; error AS vs. correct PS 0.03 greater, *p* < 0.0001).

### Relative hypometria in antisaccades

A good fit was obtained for the LMM modeling the square root of saccadic amplitude (residual skewness 0.4, excess kurtosis 1.55).

There were significant effects of task (*F*_2,9706_ = 110, *p* < 0.0001) and group-by-task interaction (*F*_2,9706_ = 2.63, *p* < 0.0001).

*Post-hoc* comparisons showed that the amplitude of correct antisaccades was greater than both AS errors and correct prosaccades in both patients and controls (*p* < 0.0005 two-tailed after Bonferroni correction in all comparisons), but did not detect a difference between erroneous and correct prosaccades in either group.

**Figure 4:**
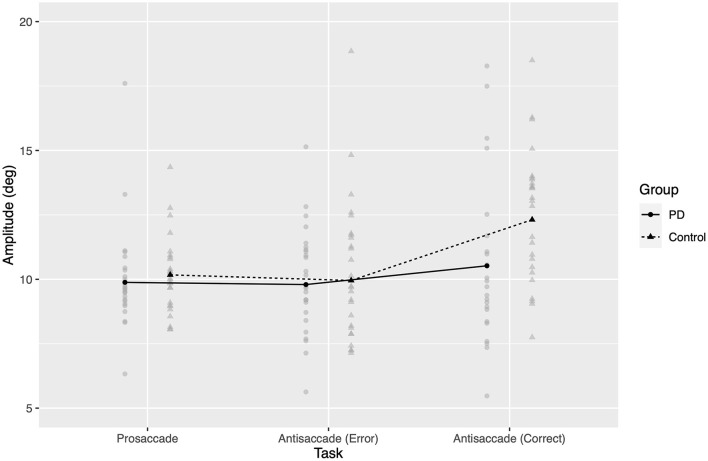
Amplitude of saccadic response, back-transformed from model of square root of amplitude. Gray points represent amplitude for one participant in a given task. Black points and lines represent estimated marginal group means.

As seen in Figure 4, PD patients did not exhibit a global hypometria (no significant fixed effect of group, *F*_1,49.6_ = 2.63, *p* = 0.11), but instead had a *relative* hypometria in correct antisaccades, where controls overshot the target location and patients did not (back-transformed control mean 12.31°, PD mean 10.52°). Back-transformed 95% confidence intervals for estimated marginal means included 10 deg in all cases except for correct antisaccades from healthy controls.

### Antisaccadic error rate

The antisaccadic directional error rate did not differ between groups (Mann–Whitney test, *U* = 243.5, *n*_PD_ = 25, *n*_HC_ = 26, *p* = 0.13two-tailed).

### Classification performance and feature rankings

**Figure 5:**
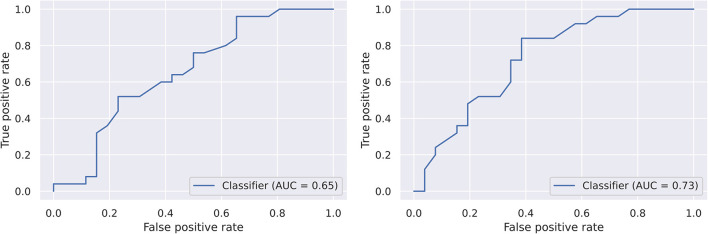
Receiver operating characteristics of the two models on the dataset generated by TLPO-CV. *Left*, logistic regression. *Right*, random forest.

The logistic regression classifier achieved ROC-AUC=0.65, and the random forest classifier achieved ROC-AUC = 0.73 in TLPO-CV (Figure 5). There was no difference in performance at the 0.05 level of significance (5 × 2CV combined *F-*test; *F* = 4.27, *p* = 0.06). However, this test reduces the size of training datasets far more than TLPO-CV, so a subsequent analysis on a larger dataset should be conducted to establish whether random forest model outperforms the logistic regression model.

**Figure 6:**
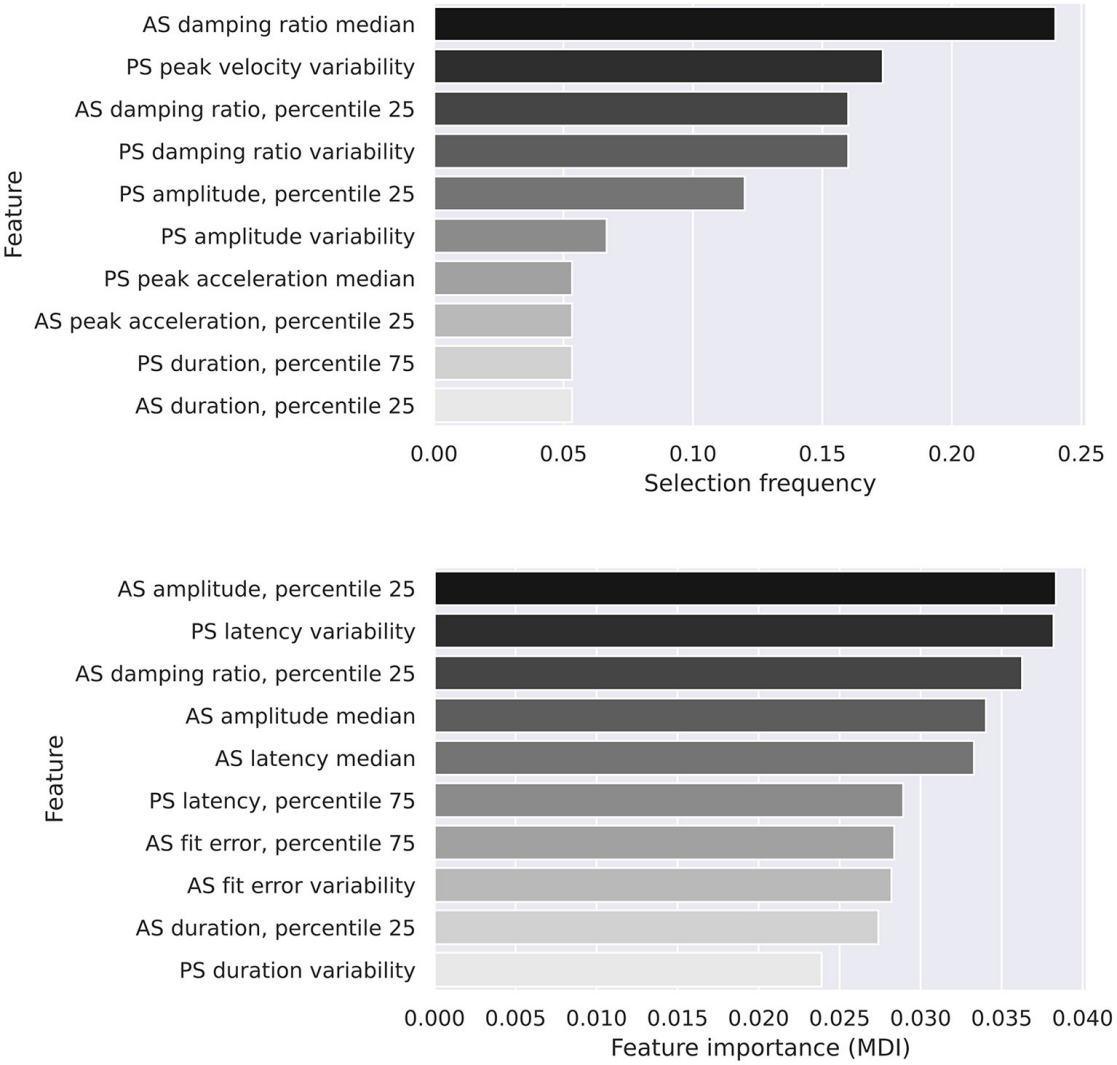
The top 10 features in each classifier. *Top*, logistic regression. *Bottom*, random forest.

While features selected by the logistic regression model were variable, both models recognized features of the antisaccadic damping ratio as important discriminators between groups (Figure 6).

## Discussion

This is a proof-of-concept study evaluating saccadic metrics, measured in patients with PD and healthy controls. We introduce two multivariate predictive classifiers to differentiate between patients with PD and controls using only saccadometric data.

Our initial analysis, focusing on variations in saccadic metrics between groups, replicates previous findings from meta-analyses. Latency, the time between peripheral target onset and saccade initiation, increased significantly from pro- to anti-saccades in both PD and HC groups. Prosaccadic and antisaccadic latencies in the PD group were both increased compared to those of HC, an established finding in published literature (5, 6). Our results do not show evidence of saccadic hypometria in PD patients compared to controls except in erroneous antisaccades, despite several previous studies having identified this (7, 28, 29). The reasons for this will be investigated in a future study and compared against video oculography to assess the possibility of calibration inconsistencies.

We evaluated the performance of two machine learning models on our data. The top 10 most important features for both the logistic regression and random forest models are shown in Figure 6. There is a degree of overlap, with quantiles of the antisaccadic damping ratio being the most frequently selected feature in the logistic regression model and the 3rd most important in the random forest model. A study by Chen et al. (13) identified damping ratios as a sensitive variable to differentiate between PD and HC groups. However, this has not commonly been calculated or studied in interim studies. This study adds to the currently small body of evidence suggesting damping ratios (calculated as per subsection Damping ratio estimation) can be used to help differentiate between patient groups, in this case in the antisaccadic task. To our knowledge, this study is the first to describe task-related changes in oculomotor damping, and suggests that the effects of PD on this phenomenon should be interpreted in context of the task. We did not replicate the effect observed by Chen et al. (13) in prosaccades at the 0.05 level of significance. Aside from the use of conservative multiple comparisons corrections in our study, there are a number of factors that could contribute to this. Firstly, the infrared oculography equipment used here has an average linearity error of 1.4° over the ±15° range within which our tests took place, which may affect measured damping ratios directly, and had a sampling rate 2.5 times lower than the electro-oculography system described in the previous study. Secondly, recording was terminated 20 ms after the velocity dropped below 5°/s, which prevented late oscillations (visible for c. 100 ms beyond this point in the data presented by Chen et al.) from contributing to our calculation of the damping ratio.

Further analyses on longitudinal datasets will allow us to investigate the possibility of using damping ratios to assess disease progression and the effects of aging, as is indicated given the cross-sectional analysis by Chen et al. (13). Specifically, antisaccadic damping should be investigated longitudinally in PD patients to strengthen the evidence and further characterize these effects. Future analyses using feature selection algorithms in machine learning models also have the potential to identify additional kinematic parameters which could be more sensitive for disease differentiation than those traditionally used, including generalized fatigue-like effects which cause long-term intra-individual variability during the performance of a task (30). Additionally, future studies should consider similar parameters in vertical as well as horizontal saccades, as pathology affects the two systems differently and results from horizontal saccades may not necessarily generalize (31, 32).

More important than single features alone are the combination of features used to predict disease status. Both models used a multitude of features ranging from amplitude and acceleration to duration and fit error (in both pro- and anti-saccades) as input discriminators. The recently developed TLPO-CV method used to estimate the ROC-AUC of the classifiers has not yet been shown to generate accurate confidence intervals, so a 5 × 2 cross-validation combined *F*-test was used to calculate differences between the models, and did not find that the difference in performance between the two models was significant. However, caution should be taken given the small effective sample sizes in this cross-validation method, and it is possible that future analyses will show that the random forest algorithm outperforms logistic regression.

Standardization amongst experimental conditions remains central to the notion of reproducible science. Saccadic metrics, due to the rapid nature of movement within a noisy environment, can often be weak and have complex relationships with experimental conditions. Though internationally recognized protocols exist to measure specific saccadic metrics (for example antisaccadic error rates), novel methodologies such as those outlined in this study, can help in two ways.

Firstly, multivariate analyses enable a more precise identification of non-linear patterns of deviation amongst saccadic metrics. Secondly, the high-resolution measurement of a saccadic trajectory would allow a larger number of informative features to be extracted from each movement. This raw data could also be present but not yet analyzed within existing datasets, allowing new metrics to be compared to existing metrics under identical experimental conditions, minimizing inter-study biases.

As shown in this study, the damping ratio was an important metric in both logistic regression and random forest models. These results were in keeping with a small cross sectional study from 1998 (13). Recent evidence suggests that PD comprises a more heterogeneous set of phenotypes than previously hypothesized (33). Metrics such as the damping ratio have the potential not only to help differentiate between PD and HC with greater accuracy, but additionally to allow for more precise definitions of differing clinical phenotypes amongst those with PD. This has numerous clinical benefits, such as the ability to offer tailored, phenotype-dependent treatment regimes, or even to shift the disease diagnosis entirely.

Saccades are easily measurable, non-invasive biomarkers which have been investigated for nearly two centuries (34). Using multivariate analyses on pre-existing, and future, datasets enables researchers to transform these data into robust and objective clinical decision tools. This in turn will benefit the diagnosis of patients, the tracking of disease progression and the monitoring of treatment effectiveness.

This pilot study outlines a novel methodology to analyze saccades, laying the foundation for future studies using larger datasets to offset current limitations imposed by the intrinsic low signal-to-noise ratio. Studies applying machine learning techniques to larger datasets have the potential to both establish a hierarchy of significance of known metrics, and to use unsupervised learning techniques to identify novel saccadic metrics hidden within trajectories.

We plan future analyses using additional machine learning approaches to try to differentiate between clinical phenotypes of PD. Unsupervised clustering approaches may shed light on distinct clinical phenotypes, which may well be a limiting factor in the performance of current models. We hope that analyses such as these will allow us to identify and stratify patients by their clinical phenotype in a more precise, accessible, and reproducible manner, improving the feasibility of advancing candidate treatments through clinical trials. Future analyses, focusing on inter-group variability, would enable researchers to investigate this in detail, adjusting for saccadic metric, current group status and additional variables such as age of onset, medication benefit, and disease course.

## Data availability statement

The original contributions presented in the study are included in the article/supplementary material, further inquiries can be directed to the corresponding author.

## Ethics statement

The study has been approved by the Research Ethics Committee (Research Ethics Committee reference 16/SW/0262). The patients/participants provided their written informed consent to participate in this study.

## Author contributions

OB, SP, CAA, and JJF designed and oversaw the study. SP and CAA oversaw the testing and implementation of the saccadic task. JJF and CAA oversaw the statistical analysis for the study. All authors critically reviewed various versions of the manuscript. All authors have seen and have access to the whole dataset presented here.

## Funding

CAA was supported by grants from the NIHR and UCB. JJF was supported by the National Institute for Health Research (NIHR) Oxford Biomedical Research Centre (BRC).

## Conflict of interest

The authors declare that the research was conducted in the absence of any commercial or financial relationships that could be construed as a potential conflict of interest.

## Publisher's note

All claims expressed in this article are solely those of the authors and do not necessarily represent those of their affiliated organizations, or those of the publisher, the editors and the reviewers. Any product that may be evaluated in this article, or claim that may be made by its manufacturer, is not guaranteed or endorsed by the publisher.
